# Grinding Force and Surface Formation Mechanisms of 17CrNi2MoVNb Alloy When Grinding with CBN and Alumina Wheels

**DOI:** 10.3390/ma16041720

**Published:** 2023-02-19

**Authors:** Xiaoyang Jiang, Ke Liu, Mingda Si, Maojun Li, Pan Gong

**Affiliations:** 1 State Key Laboratory of Advanced Design and Manufacture for Vehicle Body, Hunan University, Changsha 410082, China; 2 Jianglu Machinery & Electronics Group Co., Ltd., Xiangtan 411100, China; 3 State Key Laboratory of Materials Processing and Die & Mold Technology, School of Materials Science and Engineering, Huazhong University of Science and Technology, Wuhan 430074, China

**Keywords:** grinding force, surface morphology, material removal mechanism, defects

## Abstract

The 17CrNi2MoVNb alloy is widely used for manufacturing heavy-duty gears in vehicles’ transmission systems, where grinding is a significant process affecting gears’ working performance and service life. This work comprehensively analyzed the grinding force, surface morphology, and surface roughness when grinding 17CrNi2MoVNb alloy using alumina and CBN grinding wheels. Results showed that the maximum normal grinding force from the CBN wheel was only ~67% of the one from the alumina wheel. Due to the small size and limited cutting depth of CBN grains, the grinding force increased by about 20% when the grinding depth increased from 0.02 to 0.03 mm for CBN grinding wheels. Surface defects, including cavities and material tearing, were mainly found on the ground surface when using an alumina grinding wheel. The surface roughness Ra recorded from the CBN grinding wheel mainly ranged from 0.263 to 0.410 μm, accounting for less than 40% of the one from the alumina grinding wheel. The information from this work could provide benchmark data and references for optimizing grinding tools and parameters when manufacturing gears in the vehicle industry.

## 1. Introduction

Heavy-duty gear is an important part of the transmission system of large vehicles. Due to its harsh working environment and heavy load, cracks and other defects usually occurs on the working surface. Heat treatment is a common method to improve the performance of heavy-duty gear by increasing the hardness of the tooth surface. As the last processing procedure, grinding is used to eliminate the deformation caused by heat treatment. Thus, it mainly determines the dimensional accuracy and final surface quality of the gear component. Previous researchers proposed different methods to improve the ground surface quality, including optimizing the grinding parameters [1], promoting cooling conditions [2], using ultrasonic vibration grinding or corrosive grinding [3,4], and setting up model prediction [5]. The key point is to study the dynamic evolution of material microstructure during grinding and its impact on surface integrity.

The material and hardness of the grinding wheel are also important factors affecting ground surface quality, which are mainly manifested in different grinding force ratios and material removal mechanisms. Alumina is a commonly used grinding wheel abrasive in the mechanical production process. The variable composition content and hardness of alumina grinding wheel has a significant influence on the grinding performance of different materials as reported by previous literatures. The results of works on the grinding behavior of zircon corundum, white corundum and brown corundum wheels showed that the grinding efficiency of white corundum wheels was higher than that of brown corundum wheels, and the grinding efficiency of zirconium corundum wheels was the lowest. Still, the zirconium corundum wheels obtained superior grinding surfaces [6]. Zhang et al. [7] studied the ground surface integrity and material removal mechanism of rails using three grinding wheels with different abrasive grit sizes and claimed that the grinding mechanism transformed from rubbing and plowing to cutting operation when the grit size decreased. More effective cutting edges were generated on the grinding wheel with finer abrasives, resulting in lower surface roughness and residual stress. Wang et al. [8] conducted a grinding experiment using grinding wheels with four hardness levels and different contents of zirconium corundum and brown fused alumina abrasives. It was reported that using zirconium corundum wheels achieved a larger grinding amount, higher surface roughness, and less grinding burn. The ground surface roughness and residual stress showed decreased trends with the increase in grinding wheel hardness. Li et al. [9] studied the influence of abrasive tool wear on ground surface characteristics and corrosion resistance performance during grinding superalloy with white alumina (WA) and microcrystalline alumina (MA) wheels. It was found that the MA wheel presented lower wheel wear and higher self-sharpening than the performance of the WA wheel. Compared to the WA wheel, the MA wheel had less material adhesion, which led to a better surface finish. Dang et al. [10] studied the feasibility of creep feed grinding of ultra-high strength steel with hard zirconium corundum grinding wheel by analyzing the response of grinding force, grinding specific energy, and surface integrity. It was found that the used zirconium corundum wheel showed a higher material removal rate and compressive residual stresses and presented less surface burn and wheel wear. It can be concluded that alumina grinding wheels are wildly used in metal grinding because of their variety of species and relatively low cost. However, its low hardness limited the use of alumina wheels in grinding hardened materials.

Among the commonly used grinding wheel materials, CBN abrasive grains have higher chemical stability and superior thermal conductivity and durability than alumina abrasives. Thus, it has the potential as the preferred grinding wheel material to improve the surface quality and grinding efficiency. The grinding performance of alumina and CBN grinding wheels is still required for further exploration [11]. Yao et al. [1] studied the influence of wheel materials on grinding force when grinding ultra-high strength steel using single alumina, white alumina, and CBN wheels. Results showed that the minimum and maximum grinding force ratio could be obtained using the CBN wheel and single alumina wheel. Denkena et al. [12] reported that a nickel coating of CBN grains enhanced the embedding of grains in the bond compared with the uncoated grains. Bhaduri et al. [13] studied the grindability of low-carbon steel using monolayer brazed CBN, white, and grey alumina wheels under three cooling conditions. They found that the CBN grinding wheel outperformed the alumina wheels in terms of grinding forces, and it was the most wear resistant when cooled by pure oil. Sato et al. [14] reported that in the minimal quantity of lubrication grinding experiments, the CBN grinding outperformed the alumina grinding trials, showing a superior performance not only in terms of better surface finishing, lower surface roughness, and higher dimensional accuracy but also higher tool efficiencies, lower diametrical wear, acoustic emission, and grinding power. Zhao et al. [15] developed new CBN grinding wheels with a porous metal bond to improve tool performance and machined surface integrity. Results showed that a stable grinding force ratio and smoother ground surface were obtained due to the combined characteristics of microfracture and partial macro-fracture of CBN particles.

From the above literature, few researchers worked on the influence of different grinding wheel materials on grinding performance and surface formation mechanisms. In this work, surface grinding trials on carburized and quenched 17CrNi2MoVNb alloy were carried out using both alumina and CBN wheels, and the influence of grinding parameters on grinding force and surface roughness was also analyzed. The research work aimed to study the influence characteristics/mechanisms of two different grinding wheels on ground surface morphology and surface roughness and to further understand corresponding material removal mechanisms during the grinding process. The results can provide information for optimizing/selecting grinding wheels and parameters when grinding heavy-duty gears in vehicle industries.

## 2. Experimental Works

### 2.1. Materials

The 17CrNi2MoVNb alloy is mainly selected for manufacturing heavy-duty gears, and its chemical composition and mechanical properties are shown in Table 1 and Table 2, respectively. Because the addition of the microalloying element Nb hinders the growth of austenite grains during carburizing, the grain size of 17CrNi2MoVNb is relatively fine and uniform [16]. A small amount of Ni can improve the ductility of alloys, the added Mo is helpful for precipitation strengthening, and a small amount of V can inhibit the abnormal growth of austenite grains [17,18]. Therefore, 17CrNi2MoVNb alloys present the advantages of relatively high surface hardness as well as superior strength and toughness.

### 2.2. Experimental Setup

**Figure 1:**
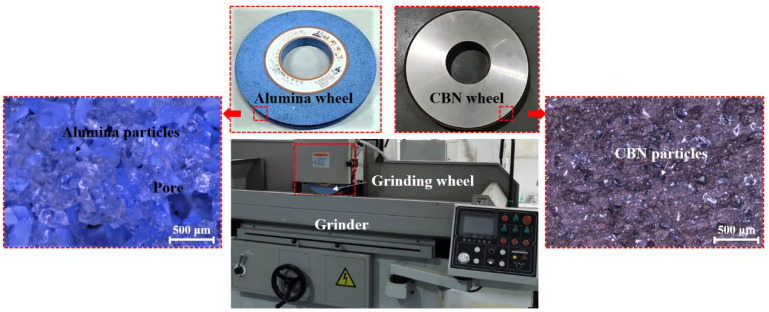
Alumina and CBN grinding wheels used in the experiment.

As shown in Figure 1, the grinding experiment of 17Cr2Ni2MoVNb workpieces was conducted on a DY-510ASM automatic hydraulic high-precision grinder, of which the maximum output power was 7.5 kW. As the alumina grinding wheel was used in the production line of the corresponding product in this study, it was employed in the grinding trials as the benchmark. The workpiece in this study was a 17CrNi2MoVNb alloy with high hardness after carburizing and quenching processes, so it was feasible to use a CBN grinding wheel for grinding with high efficiency and good surface quality. A SiC-based grinding wheel was mainly used for grinding metallic materials such as cast iron and non-ferrous metals as well as non-metallic materials. Thus, both CBN and alumina grinding wheels were selected for the grinding experiment. An alumina grinding wheel with grain sizes of 150–180 μm and a CBN grinding wheel with grain sizes of 90–125 μm were applied. The diameter of both grinding wheels was 350 mm, and the width was 34 mm. 17CrNi2MoVNb alloy was cut into small samples with the dimensions of 30.0 mm × 34.0 mm × 15.0 mm. Two levels of grinding depth, three levels of grinding speed, and feed speed were employed. The detailed grinding parameters are shown in Table 3.

**Figure 2:**
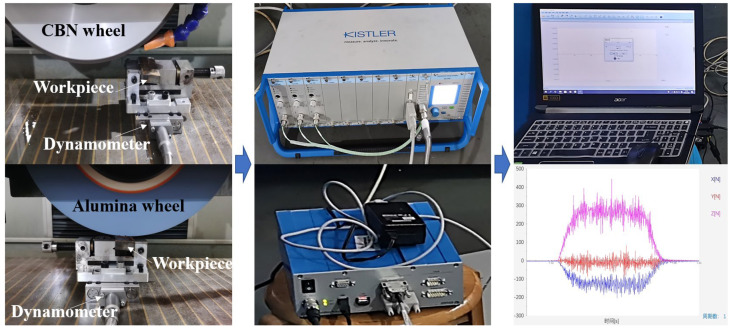
Grinding force measuring devices for grinding experiment.

The ground surface topography was observed by an optical microscope, and the ground surface roughness was measured by a TR 200 roughness tester; 36 samples were analyzed, and each one was repeated three times, then the average value was recorded. The measuring direction was perpendicular to the abrasive cutting one. The ground workpieces were cut into small pieces with the size of 8.0 mm × 3.0 mm × 4.0 mm and mounted with resin for SEM and EDS observation on an EM-30N ultra-high resolution scanning electron microscope (COXEM EM-30). All the samples were polished and corroded by 4% nitric acid alcohol reagent for 15 s. A KISTLER (9119AA2) dynamometer was used to measure the grinding force, as shown in Figure 2.

## 3. Results and Discussion

### 3.1. Grinding Force Analysis

**Figure 3:**
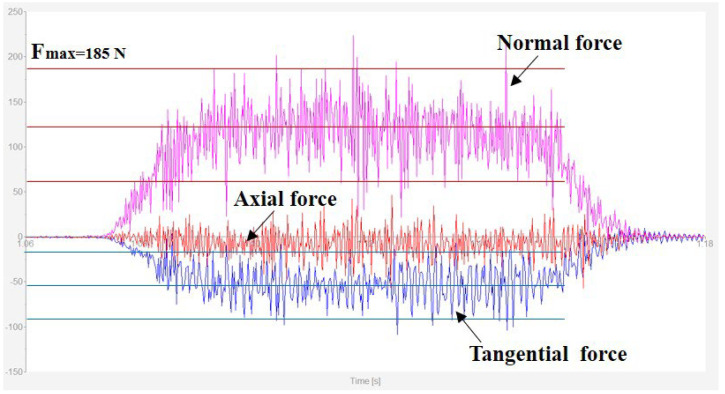
Typical grinding force profile recorded from the measurement system.

**Figure 4:**
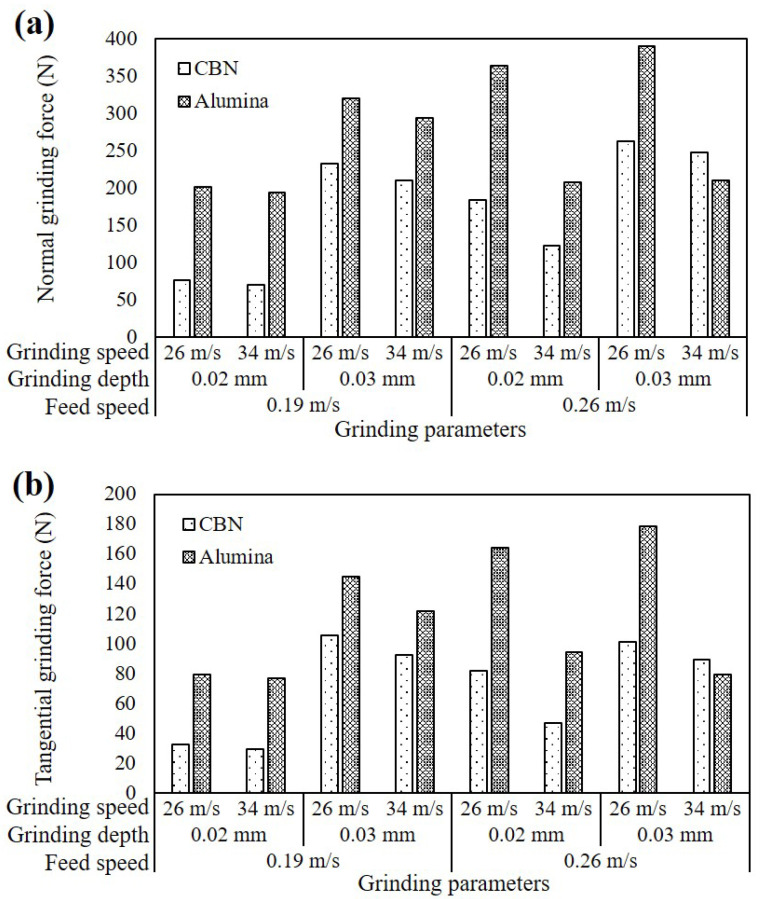
(**a**) Normal grinding force and (**b**) tangential grinding force at different grinding parameters using the CBN and alumina grinding wheels.

The typical grinding force profile collected by the dynamometer is shown in Figure 3. It can be seen from the curve that normal force and tangential force increased when the grinding wheel started contacting the workpiece. When grinding was in a stable stage, the grinding force level fluctuated within a certain range. Eventually, the grinding forces decreased to zero when the pass was finished. The axial force, which was mainly related to the vibration of the grinder, fluctuated on the zero-horizontal line, which will not be discussed in this paper. The maximum value of tangential force and normal force was selected for further analysis. The grinding forces from each set of grinding parameters are detailed in Figure 4. The normal grinding force of the CBN wheel ranged from 70 N to 260 N, accounting for 36% to 67% of that from the alumina wheel. The top value of the tangential force of the alumina wheel was 178 N, 70% higher than the one recorded from the CBN wheel. It was obvious that whether the CBN wheel or alumina wheel, when the grinding depth increased from 0.02 mm to 0.03 mm, the increase of normal force and tangential force was almost more than 20%. The increased grinding depth contributed to an increase in material removal rate, which indicated that the cutting workload of each grain increased, leading to an increase in grinding force [19]. However, for the alumina wheel, at the feed speed of 0.26 m/s and the grinding speed of 34 m/s, the increase of grinding depth showed a less significant effect on grinding force. This was possible because of the thermal softening of the material surface caused by severe friction and plastic deformation. Work hardening might lead to the increase of grinding force, while due to the relatively lower thermal conductivity of the alumina grinding wheel, thermal softening became more obvious and promoted the decrease of grinding force [19]. The influence of thermal softening on the grinding force was opposite to that of increasing material removal rate. Both of them occurred at the same time, resulting in a small variation of grinding force. The grinding force tended to increase with the increase of feed speed, as the amount of material removed per unit of time and the grinding thickness of each abrasive particle increased [20].

CBN grinding wheel has the superiority in generating a lower level of grinding force than the alumina grinding wheel. At the grinding depth of 0.02 mm, the grinding force of the CBN wheel was 50% or even lower than that of the alumina wheel, but when the grinding depth increased to 0.03 mm, this proportion increased to more than 70%. It meant that the grinding force difference between the CBN wheel and the alumina wheel was reduced with such a group of grinding parameters. When the grinding depth was 0.02 mm, the grinding force was lower because the sharpness of the CBN grinding wheel was higher, and the cutting advantage was more obvious. At the grinding depth of 0.03 mm and the feed speed of 0.26 m/s, when the grinding speed increased from 26 m/s to 34 m/s, it was obvious that the grinding force of the alumina wheel decreased with grinding speed more than that of the CBN wheel. This was possibly due to the poor thermal conductivity of the alumina grinding wheel generating the thermal softening effect.

**Figure 5:**
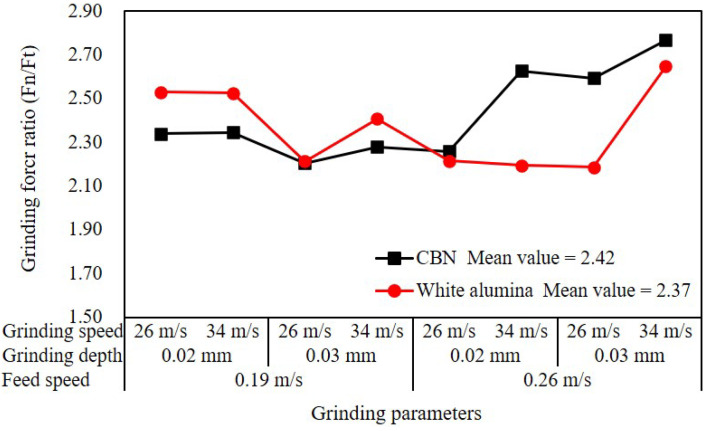
Variation of force ratio with different grinding parameters.

The variation of the force ratio of two grinding wheels at different grinding parameters is shown in Figure 5. The force ratio was in the range of 2.19~2.77, and the mean value of the CBN wheel and alumina wheel was 2.42 and 2.37, respectively. The grinding force ratio of 17CrNi2MoVNb was less than 3.00, indicating that the material was more brittle than other common alloys due to the carburizing and quenching treatments before grinding [20]. It was worth noting that the force ratio of the CBN wheel showed a limited increase when the grinding depth increased to 0.03 mm, possibly because the small cutting depth of CBN grits was difficult to increase the material removal rate, which led to the increase in of normal force. It can be observed from the red curve that the force ratio of the alumina wheel increased when the grinding speed increased from 26 m/s to 34 m/s at the grinding depth of 0.03 mm and the feed speed of 0.26 m/s. It was mainly attributed to the thermal effect caused by friction and plastic deformation. When considering the characteristic of grinding wheel material, it can be found that the self-sharpening effect of alumina wheels at large grinding depths may also contribute to the increase of force ratio [21].

### 3.2. Microstructure Evolution within Ground Surface Layer

**Figure 6:**
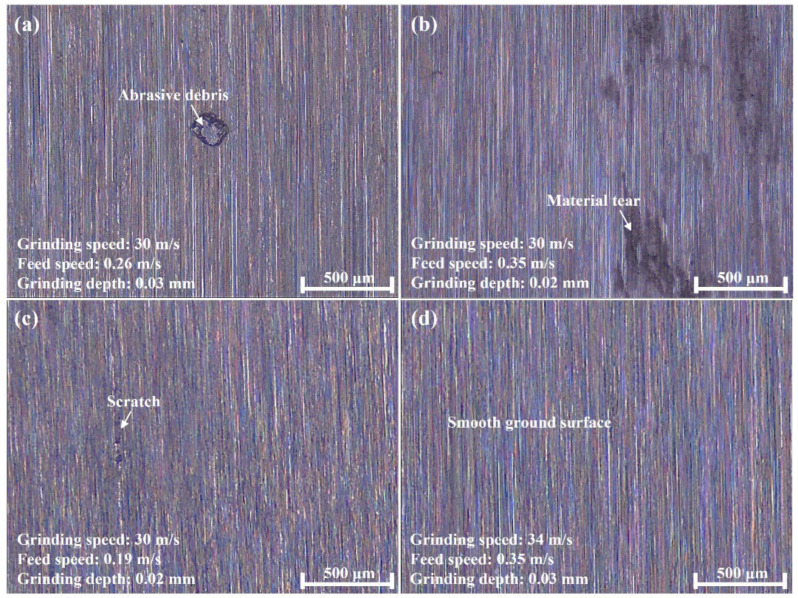
Typical surface morphology generated by an alumina wheel at (**a**) 30 m/s, 0.26 m/s and 0.03 mm, (**b**) 30 m/s, 0.35 m/s and 0.02 mm, and a CBN wheel at (**c**) 30 m/s, 0.19 m/s and 0.02 mm, and (**d**) 34 m/s, 0.35 m/s, and 0.03 mm.

**Figure 7:**
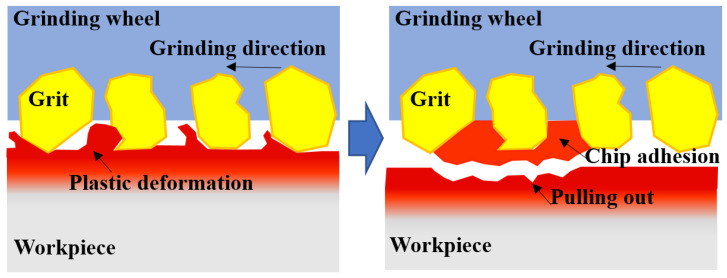
Schematic showing material pulling out on the ground surface.

Figure 6 shows the ground surface morphology from typical grinding trials. Defects such as grain embedding and surface potholes were observed on the surface ground by the alumina wheel, while the ground surface from the CBN wheel was relatively smooth. The dense scratches on the surfaces ground by the two wheels were in the same direction with uniform scratch depth, and few deep grooves could be found obviously. Although no cracks were observed, the material tearing was found in Figure 6b, and the formation mechanism of material tearing is illustrated in Figure 7. It was possibly caused by the adhesion characteristics and wear behavior of the alumina material, together with relatively high grinding temperature and severe plastic deformation in the grinding area. The surface material that adhered to the wheel surface was pulled out [9].

**Figure 8:**
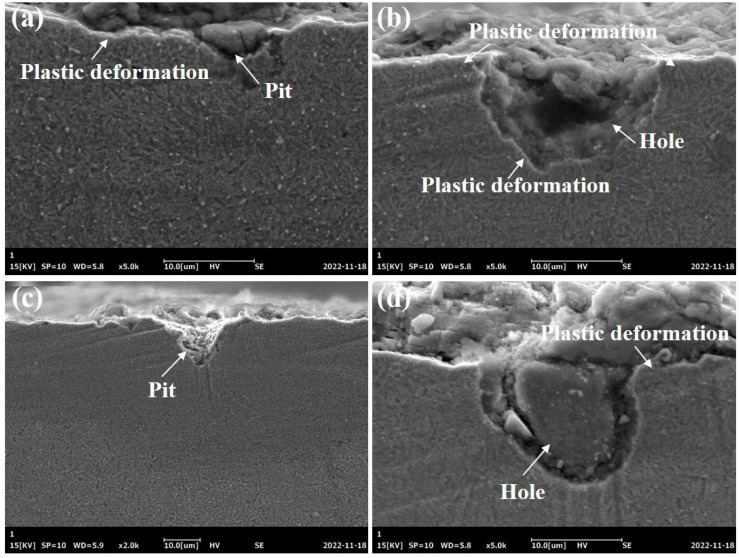
SEM images of defects on grinding surface when using an alumina wheel (**a**,**b**) at 0.19 m/s, 0.02 mm, and 26 m/s, (**c**,**d**) at 0.19 m/s, 0.02 mm, and 34 m/s.

**Figure 9:**
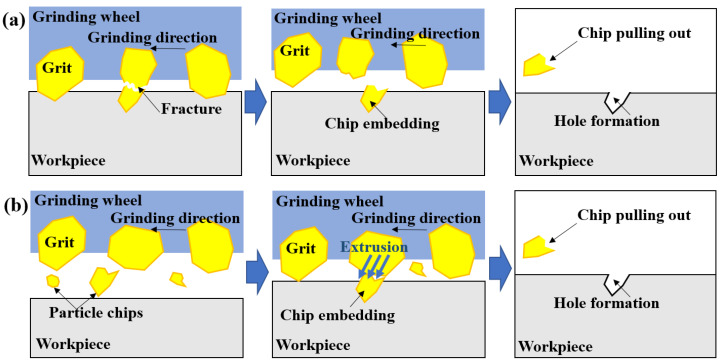
Schematic showing formation mechanism of pits on the ground surface including (**a**) abrasive particle broken in the workpiece, (**b**) abrasive particle fractured out of the ground surface and squeezed in the workpiece.

Figure 8 shows the SEM morphology of the subsurface generated by the alumina grinding wheel. Pits observed in the figure were the common defects. The grain size of the alumina grinding wheel was between 150 μm and 180 μm, while the size of the pits exceeded 10 μm but no more than 20 μm. Figure 9a presents a typical mechanism for producing such pits. The alumina grain with a large cutting depth broke, and the chips were embedded in the workpiece. When the embedded abrasive chips fell off while polishing the sample, such holes were left on the interface [22]. Another possible reason is shown in Figure 9b, the alumina abrasive grains fractured due to thermal softening and large grinding force during the grinding process, and the debris of the abrasive grains was pushed into the ground surface by the grinding wheel [8]. This kind of defect was based on the poor thermal conductivity and adhesion characteristics of the alumina grinding wheel.

**Figure 10:**
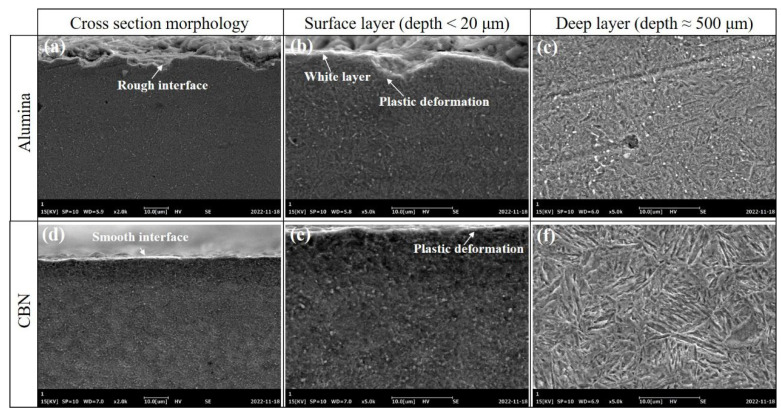
Subsurface morphology after grinding at a feed speed of 0.19, grinding depth of 0.02 mm, and a grinding speed of 26 m/s with (**a**–**c**) alumina wheel, (**d**–**f**) CBN wheel.

Figure 10 shows SEM images of the ground subsurface and related material morphology. By comparing the cross-section profile of the ground surface, it can be found that the CBN wheel grinding surface profile was smooth, while the alumina wheel grinding surface profile was rough, including short lines, bumps, and pits. In addition to the pits, it can also be observed that a bright white dense layer on the surface ground by the alumina wheel, which was similar to the plastic deformation layer caused by mechanical extrusion [23]. The surface material was crushed into smaller grains under the extrusion of the grinding wheel, so the structure became relatively dense. It was found that there was also a thin plastic deformation layer on the surface ground by the CBN grinding wheel, and the grain morphology in the surface layer was difficult to recognize, which was probably due to grain refinement.

**Figure 11:**
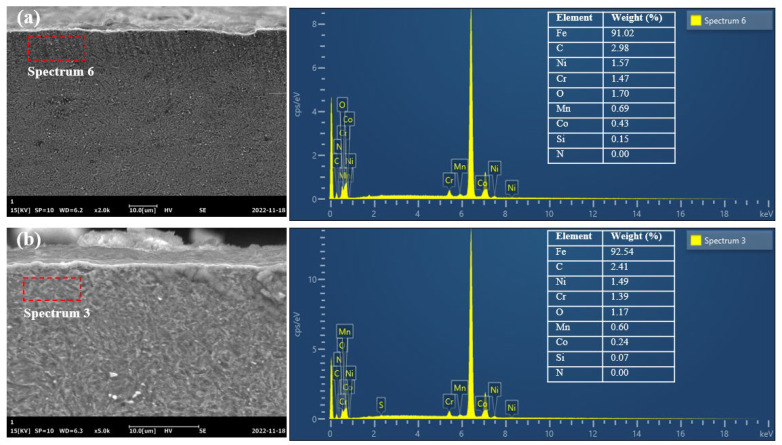
EDS spectra at the subsurface layer after grinding using (**a**) an alumina wheel and (**b**) a CBN wheel at a feed speed of 0.26 m/s, grinding depth of 0.03 mm, and grinding speed of 30 m/s.

Figure 11 presents the EDS spectrum of surface layer ground by different grinding wheels. By comparing the change of oxygen content on the grinding surface, the influence of grinding parameters on the oxidation of the material surface in the grinding process could be analyzed [24]. The oxygen content of the ground surface with the alumina wheel was 1.70%, while the one with the CBN wheel was 1.17%. The CBN grinding wheel obtained lower oxygen content on the ground surface, which might be related to the superior thermal conductivity of CBN materials. Moreover, the CBN grinding wheel had a smaller grain size, while the alumina grinding wheel had higher porosity. Thus, the oxygen content was higher in the grinding area, leading to the grinding surface being more prone to oxidation. Grinding was essentially an abrasive-cutting process accompanied by high thermal and mechanical stress. A large amount of heat in the grinding zone was transferred to the workpiece, inducing a series of physical and chemical changes [25].

**Figure 12:**
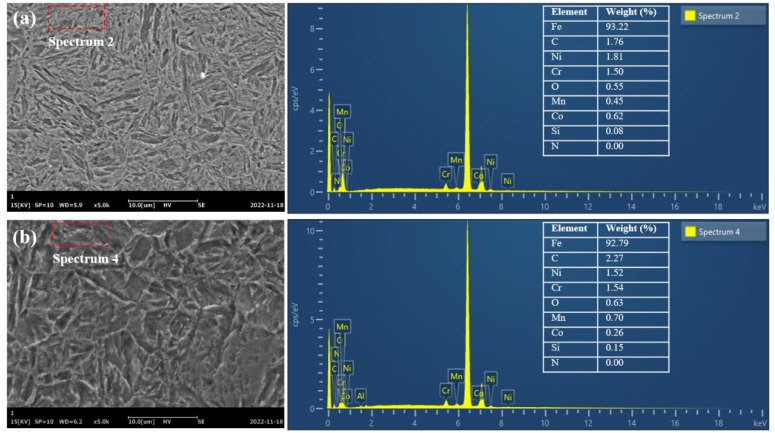
EDS spectra at a depth of 500 μm below the ground surface when using (**a**) an alumina wheel and (**b**) a CBN wheel at a feed speed of 0.26 m/s, grinding depth of 0.03 mm, and grinding speed of 30 m/s.

Figure 12 shows the EDS spectrum of deep-layer material after grinding with two kinds of grinding wheels. The 17Cr2Ni2MoVNb steel used in this experiment was carburized and quenched; thus, the carbon content in the surface layer was higher than in the deep layer, which decreased with the increase in depth. The content of carbon element at 0.50 mm below the grinding surface of the alumina wheel and CBN wheel decreased to 1.76% and 2.27%, respectively, while the content of oxygen element decreased to 0.55% and 0.62%, and the content of other elements changed slightly. The change in elements content indicated that both kinds of grinding wheels caused slight oxidation on the grinding surface. Still, there was no obvious influence on the composition and content of the carburized layer.

### 3.3. Surface Roughness Analysis

**Figure 13:**
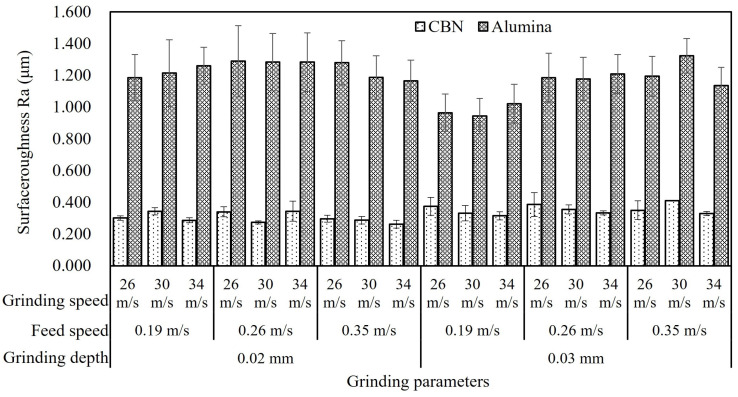
Surface roughness Ra on the vertical direction with different grinding parameters.

The surface roughness Ra obtained by two grinding wheels is summarized in Figure 13. The surface roughness ground by the CBN wheel varied from 0.263 μm to 0.410 μm, while the one from the alumina wheel ranged from 0.944 μm to 1.325 μm for all trials. It was obvious that the ground surface roughness was mainly determined by the material of the grinding wheel in this experiment. Firstly, the abrasive grain size of the CBN wheel was smaller than that of the alumina wheel. Secondly, the hardness, which was defined as the connection between particles and adhesive of the CBN wheel, was also higher. Thus, the CBN grinding wheel produced a relatively smooth grinding surface. The alumina grinding wheel had larger abrasive grains, which could generate deep scratches during the grinding process. The fracture of abrasives and the embedding of chips might result in a poor surface finish. Compared to the alumina wheel, the CBN grinding wheel had small abrasive grains and more effective cutting edges during the grinding process, which helped to improve the surface quality. Additionally, the poor thermal conductivity of the alumina grinding wheel probably also induced severe plastic deformation, thus increasing the surface roughness.

**Figure 14:**
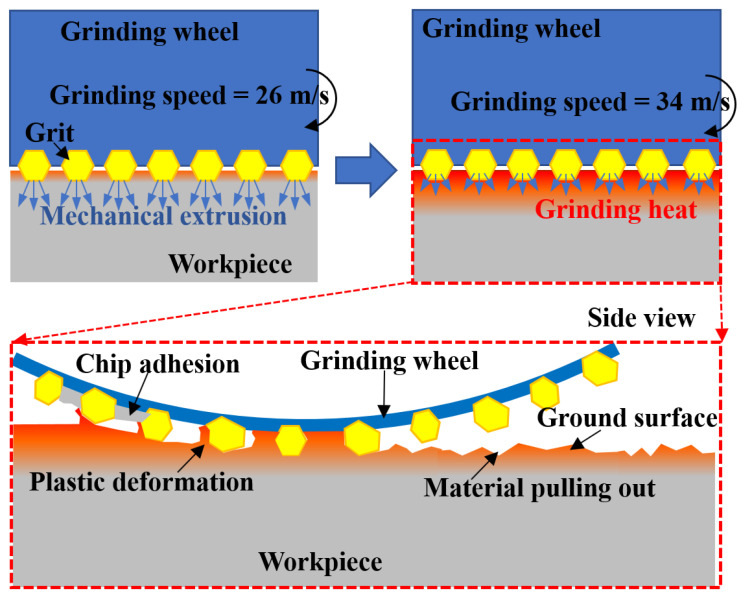
Schematic showing the mechanism of mechanical and thermal effects on surface roughness.

The maximum difference of surface roughness from the CBN grinding wheel was 0.137 μm, while the one for the alumina grinding wheel was 0.188 μm at each group parameter except for the three groups at a grinding depth of 0.03 mm and a feed speed of 0.19 m/s. It indicated that the influence of grinding parameters on surface roughness was limited when considering grinding wheel material. It was worth noting that at the grinding depth of 0.03 mm and the feed speed of 0.19 m/s, the ground surface roughness of alumina was lower than 1.00 μm, which was obviously lower than the ones obtained by other grinding parameters. It was possible because the alumina grinding wheel provided a balance between mechanical action and thermal effect at this group of parameters. The influence mechanism of mechanical and thermal effects on surface roughness is shown in Figure 14. The mechanical stress increased at a large grinding depth, and the surface became smooth due to the extruding effect of the grinding wheel. On the other hand, thermoplastic deformation was inhibited at a low feed speed of 0.19 m/s so that the material was removed sufficiently. Severe plastic deformation at high temperatures prompted chips to obstruct the pores of the grinding wheel, increasing the possibility of material pulling out, and finally, it resulted in cavities on the ground surface.

## 4. Conclusions

1. The normal force and tangential force generated by the CBN grinding wheel were up to ~64% and ~61% lower compared to the one from the alumina wheel due to the relatively higher wear resistance and thermal conductivity of the CBN wheel. The grinding force increased by about 20% when the grinding depth increased from 0.02 to 0.03 mm for CBN grinding wheels.
2. Surface defects, including cavities and material tearing, were found on the ground surface when using an alumina grinding wheel. The cavities were mainly caused by the embedding of broken abrasive grains, and material tearing resulted from plastic deformation of materials under high temperature, which was adhered to the gaps of the grinding wheel and pulled out eventually.
3. The influence of grinding wheel material on ground surface roughness was significant when compared with the effects of grinding parameters. The surface roughness Ra recorded from the CBN grinding wheel mainly ranged from 0.263 to 0.410 μm, accounting for less than 40% of the one from the alumina grinding wheel. CBN wheel presented a superior performance in grinding force and surface roughness with a feed speed of 0.19 m/s and grinding depth of 0.02 mm.
4. The future direction is suggested to evaluate the effects of abrasive size on ground surface quality, corresponding wheel wear, and material removal mechanisms. The influence of surface integrity on fatigue performance is also important for further work.

## Figures and Tables

**Table 1:** Chemical compositions of 17CrNi2MoVNb steel (wt. %) [17].

C	Si	Mn	Cr	Ni	Al	Mo	V	Nb
0.188	0.015	0.40	1.83	1.63	0.048	0.31	0.093	0.059

**Table 2:** Mechanical properties of 17CrNi2MoVNb steel (wt. %) [18].

2% YS (MPa)	UTS (MPa)	EL (%)
964~1362	1019~1456	12.48~14.80

**Table 3:** Grinding parameters when using alumina and CBN grinding wheels.

Grinding Parameters	Level 1	Level 2	Level 3
Grinding depth (mm)	0.02	0.03	
Feed speed (m/s)	0.19	0.26	0.35
Grinding speed (m/s)	26	30	34

## Data Availability

The data that support the findings of this study are available from the corresponding author upon reasonable request.
